# Mode of clinical presentation and delayed diagnosis of Turner syndrome: a single Centre UK study

**DOI:** 10.1186/s13633-018-0058-1

**Published:** 2018-06-26

**Authors:** Louise Apperley, Urmi Das, Renuka Ramakrishnan, Poonam Dharmaraj, Jo Blair, Mohammed Didi, Senthil Senniappan

**Affiliations:** Department of Paediatric Endocrinology, Alder Hey Children’s Hospital NHS Trust, Liverpool, UK

**Keywords:** Turner syndrome, Short stature, Karyotype

## Abstract

**Background:**

Early diagnosis of girls with Turner syndrome (TS) is essential to provide timely intervention and support. The screening guidelines for TS suggest karyotype evaluation in patients presenting with short stature, webbed neck, lymphoedema, coarctation of aorta or ≥ two dysmorphic features. The aim of the study was to determine the age and clinical features at the time of presentation and to identify potential delays in diagnosis of TS.

**Methods:**

Retrospective data on age at diagnosis, reason for karyotype analysis and presenting clinical features was collected from the medical records of 67 girls with TS.

**Results:**

The mean age of diagnosis was 5.89 (±5.3) years ranging from pre-natal to 17.9 years (median 4.6 years). 10% were diagnosed antenatally, 16% in infancy, 54% in childhood (1–12 years) and 20% in adolescence (12–18 years). Lymphoedema (27.3%) and dysmorphic features (27.3%) were the main signs that triggered screening in infancy. Short stature was the commonest presenting feature in both childhood (52.8%) and adolescent (38.5%) years. At least 12% of girls fulfilled the criteria for earlier screening but were diagnosed only at a later age (mean age = 8.78 years). 13.4% of patients had classical 45XO karyotype and 52.3% of girls had a variant karyotype.

**Conclusion:**

Majority of girls with TS were diagnosed only after the age of 5 years. Short stature triggered evaluation for most patients diagnosed in childhood and adolescence. Lack of dedicated community height-screening programme to identify children with short stature and lack of awareness could have led to potential delays in diagnosing TS. New strategies for earlier detection of TS are needed.

## Background

Dr. Henry Turner, an American Endocrinologist, first described Turner Syndrome (TS) in 1938. It is a relatively common chromosomal disorder affecting approximately 1 in 2500 live female births [1–3]. It is secondary to complete or partial X chromosome monosomy [1–5].

The most common aetiology is complete X monosomy (45, X) [6]. It is believed that 99% of foetuses with the classical 45, X karyotype spontaneously abort, making up 10% of all miscarriages within the first trimester [2, 4]. There are two aetiologies for partial X monosomy. The first is mosaic form, a cell line of 45, X with 46, XX and/or 47, XXX, which is a consequence of disruption in the early stages of mitosis [6]. The other aetiology is due to abnormal meiotic recombination resulting in deletion or rearrangement of the short arm of the second sex chromosome. An example of this is 46,X,i(Xq)/45,X [6], indicating that the second cell line shows an isochromosome of the second X chromosome with duplication of the long arm (q) and loss of the short arm (p).

The most common presentation for TS is short stature, but the phenotypical features vary and include ovarian, cardiovascular (e.g. coarctation of the aorta and bicuspid aortic valve) and renal disorders (e.g. duplicated or cleft renal pelvis; horseshoe kidney) [1, 2, 6]. Other features include webbed neck, broad chest with widely spaced nipples, cubitus valgus, low posterior hairline and multi-pigmented naevi [1, 2, 6]. These features tend to influence the age at which the diagnosis of TS is made. Cheung et al. [1] described that one fifth of cases were diagnosed within the neonatal period in view of the typical clinical features. One fifth of cases were diagnosed during childhood following investigation of short stature, and 50% of patients were not diagnosed until adolescence because of primary amenorrhoea [1].

The diagnosis of TS and thus intervention can be often delayed in children. Sävendahl and Davenport completed a study in 2000 which aimed to measure the delay in diagnosis of TS and to recommend a screening tool to aid earlier diagnosis [7]. The study, based in North Carolina, included 81 patients with karyotype-proven TS and showed mean (SD) age at diagnosis to be 4.2(5.6) years [7]. Overall the estimated delay in diagnosis for the patients diagnosed in the childhood or adolescent groups was 7.7 (5.4) years. Short stature was the trigger for the majority of the screening. Thus, with their results they developed proposed guidelines for the diagnosing TS [7].

In this study, we aimed to evaluate the mode of clinical presentation and identify the potential delay in the diagnosis of TS.

## Methods

Retrospective clinical data was obtained from the medical records of 67 TS patients who are currently under the care of Alder Hey Children’s Hospital, Liverpool, UK, a tertiary paediatric hospital. The data on existing TS patients was collected over a one-year period. The following data on clinical examination (performed by an endocrinologist and clinical geneticist as part of routine care) was obtained: 1) age at diagnosis of TS 2) presenting clinical features 3) reason for karyotype analysis and 4) karyotype result. To determine the potential delay, the proposed screening guidelines for TS were used [7]. These guidelines were developed from the findings of a study published by Sävendahl et al. [7].

To determine the potential delay the age at diagnosis was assessed in relation to the guidelines developed by Sävendahl and Davenport’s study in which girls with evidence of at least one of the following features required screening for TS [1. Unexplained short stature – defined as “height less than the fifth percentile”; 2. Webbed neck; 3. Peripheral lymphoedema; 4. Coarctation of the aorta; 5. Delayed puberty defined as “absence of Tanner Stage 2 breast development by age 12.5 years” [7]]. It was proposed that girls with a minimum of two of the following dysmorphic features should be screened for TS (1. Nail dysplasia; 2. High arched palate; 3. Short fourth metacarpal bone; 4. Strabismus) [7]. It was also highlighted that TS must be considered if the clinician finds other features (e.g. non- verbal learning disability, epicanthial folds, ptosis, cubitus valgus, multiple naevi, renal malformations, bicuspid aortic valve, recurrent otitis media and the need for glasses) on their examination [7].

## Results

**Fig. 1:**
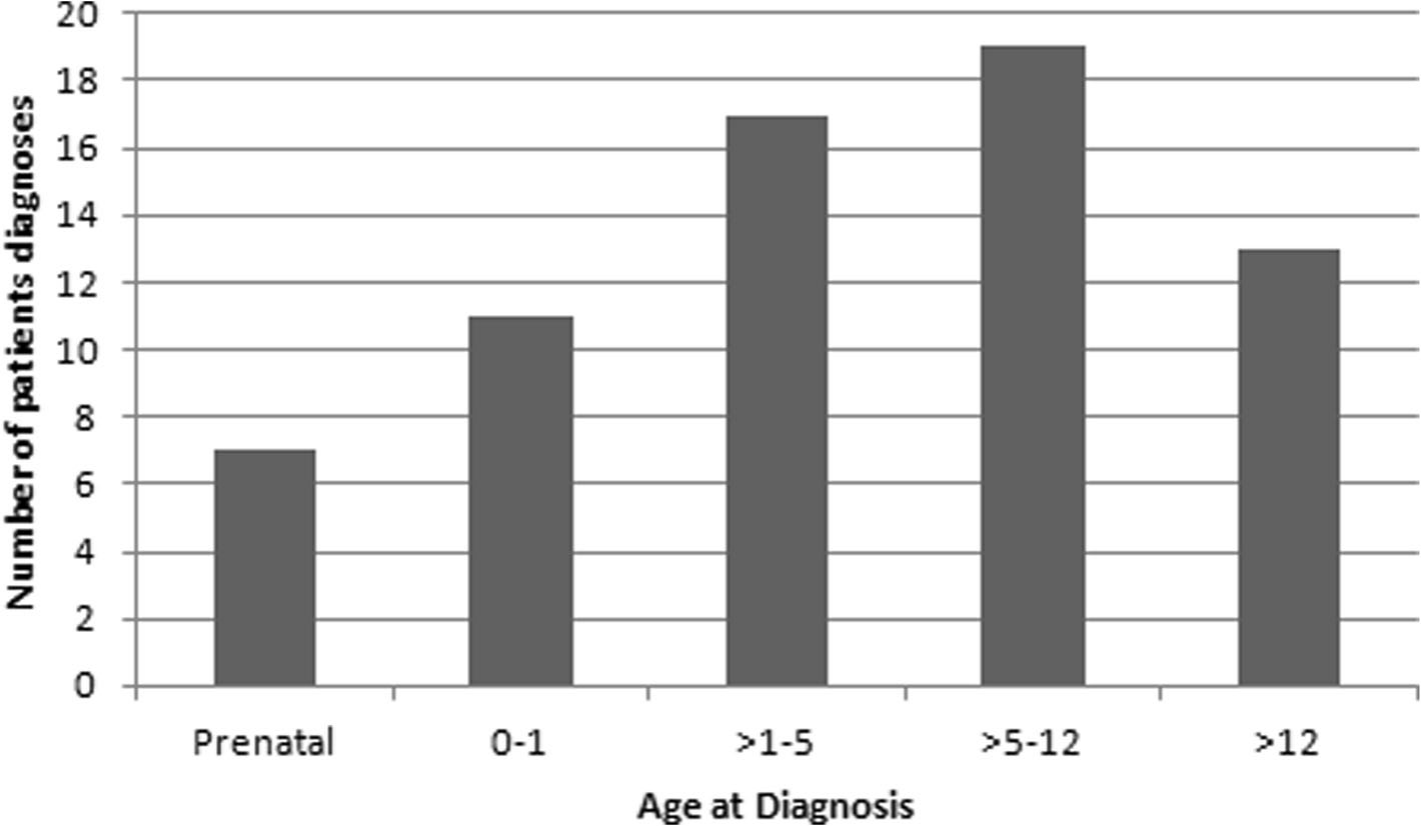
A Bar Chart that shows the age of diagnosis within our sample group

The data for this study was collected over 1 year and included 67 existing patients diagnosed with TS at the time of starting the data collection. One patient was excluded from the sample as the age of diagnosis could not be identified from the case notes. The mean age of diagnosis was 5.89 (± 5.3 years) ranging from pre-natal to 17.9 years (median 4.6 years). Seven girls (10%) were diagnosed during prenatal life, 11 (16%) were diagnosed in infancy, 36 (54%) were diagnosed during childhood and 13 (20%) were diagnosed during adolescence (Fig. 1).

### Prenatal

Of the girls who were diagnosed antenatally, 29% were screened because of increased maternal age. Unfortunately, our records could not identify the reasons for screening for the other patients. The clinical features of those diagnosed antenatally included: 1) high-arched palate; 2) neck webbing; 3) widely spaced nipples; 4) nail dysplasia and 5) low hair line.

### Infancy (birth to 1 year of age)

**Fig. 2:**
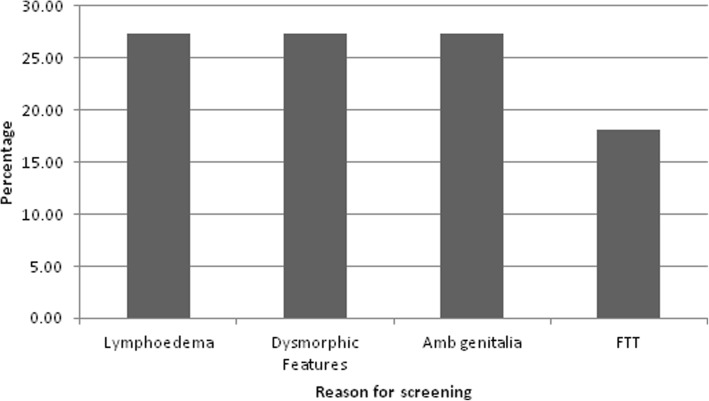
The graph shows the common reasons that patients presented in the infancy period (birth to 1 year of age) and thus diagnosed subsequently with TS *FTT = Failure to Thrive.

In total, 11 patients of the sample size were diagnosed in this age group. The mean age of diagnosis during infancy was 0.31 years (range 0–0.8 years). The main reasons for screening were: 1) lymphoedema; 2) dysmorphic features and 3) ambiguous genitalia (Fig. 2). Another presenting feature was failure to thrive.

### Childhood (> 1–12 years of age)

**Fig. 3:**
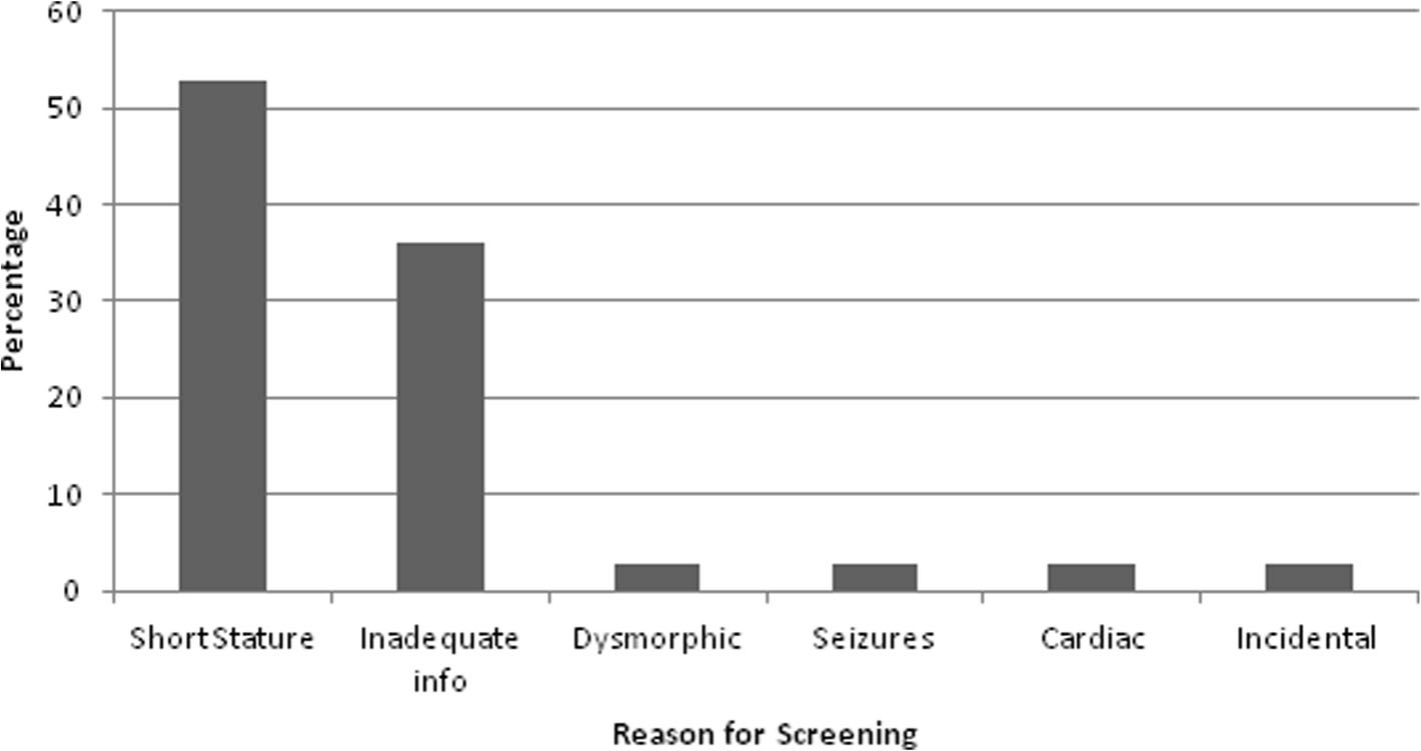
This graph shows the presenting features for children diagnosed with TS within the childhood period (> 1 year to 12 years). This shows that short stature is the main presenting complaint for patients who are thus screened for TS

53% of patients within this group were screened for TS secondary to short stature (Fig. 3). The mean age of diagnosis was 5.35 years (range 1.5–11.8 years). Interestingly, four patients were noted to have lymphoedema on examination, one had over two dysmorphic features for TS and one patient had webbing of the neck. Therefore, at least six of the 36 patients within this childhood group should have been screened at birth if the proposed guidelines had been used. The actual duration of delay in children presenting with short stature could not be ascertained due to lack of height measurements prior to seeking specialist opinion.

### Adolescence (> 12–18 years of age)

**Fig. 4:**
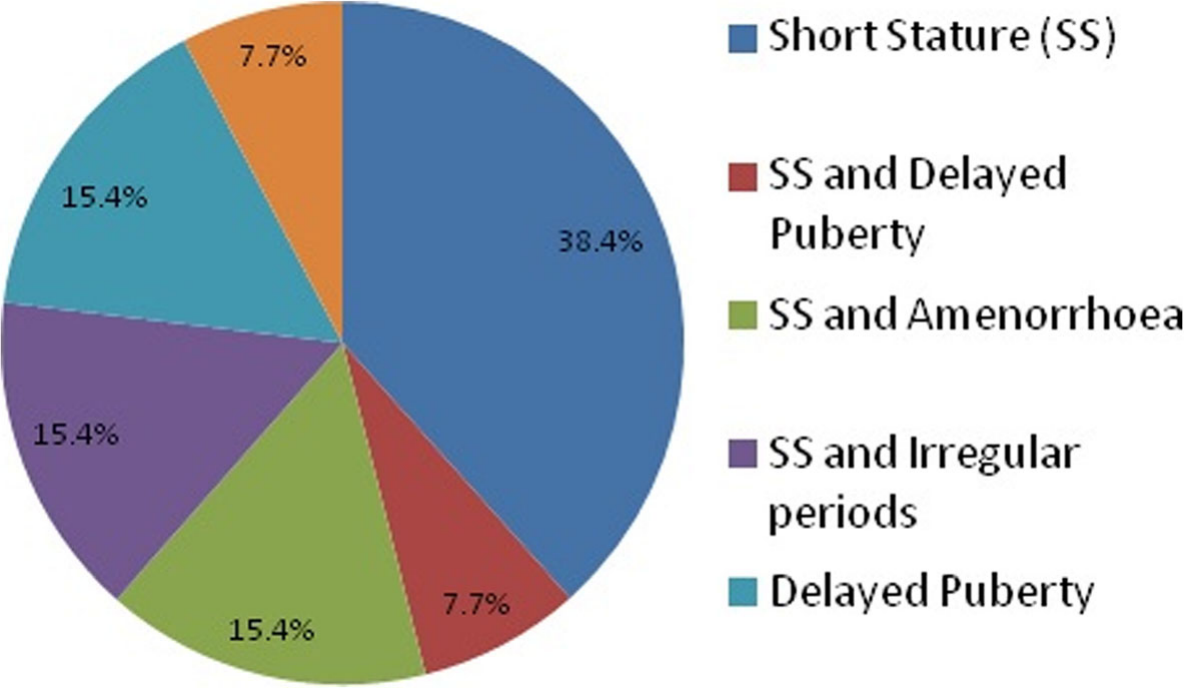
This graph shows all the reasons why patients in the adolescence period (> 12 years to 18 years) presented with before being diagnosed with TS. This suggests that short stature was the main trigger for screening. * SS – short stature

Thirteen of the girls were diagnosed during adolescence with a mean age of 15.3 years (range 12.2–17.9). The main trigger for screening was short stature (38% of patients) followed by delayed puberty (Fig. 4). Of all the patients, short stature was noted in 77% of them when examined and 38% had signs of delayed puberty. Other features noted in this group were: 1) high arched palate; 2) nail dysplasia; 3) cubitus valgus; 4) irregular periods; 5) amenorrhoea; 6) low hair line; 7) otitis media; 8) epicanthial folds; 9) widely spaced nipples; 10) wide carrying angle; 11) hypothyroidism; 12) hearing loss; and 13) renal complications. Two of the thirteen females had over two dysmorphic features and therefore, if using the proposed guidelines should have been screened at birth. This would have resulted in an earlier diagnosis of TS and potentially improved the quality of their lives.

### Genotype

The karyotype for each patient was also analysed. From the case notes, karyotypes were found for 44 of the 67 patients. Due to the retrospective nature of this study not all karyotypes could be collected but all patients had been genetically confirmed to have TS. 20.5% of these patients had classical 45X karyotype and 79.5% of girls had a variant karyotype (mosaic pattern, deletion etc.). There was no difference in the mean age of diagnosis between the girls with classical and variant karyotypes (mean age of diagnosis for both groups was 5.3 years). Table 1 identifies the types of karyotype within our sample group.

**Table 1:** Genotype of the study group (*n* = 44)

Karyotype	Number of patients (%)
45X	9 (20.5)
Mosaic (non-specified)	9 (20.5)
45X/46Xi(Xq)	4 (9)
45X/46XY	2 (4.5)
45X/46XX	5 (11.4)
45X/46XX/47XXX	1 (2.3)
XX, X, ring chromosome	1 (2.3)
45X/46XrX	3 (6.8)
45X/47XXX	3 (6.8)
Deletion of short arm of X chromosome	2 (4.5)
Deletion of long arm of X chromosome	2 (4.5)
Mosaic 45X with Y material	1 (2.3)
45XO/46XX with complex rearrangement of 2nd X chromosome including SLY gene expression	1 (2.3)
46Xi(X)(q10)	1 (2.3)

## Discussion

Earlier diagnosis of TS would help initiate appropriate management and counselling aimed to minimize long-term complications and co-morbidities. Overall, this would improve the quality of life for these patients. Massa et al. collected data, in Belgium, from 1991 to 2002 which included 242 patients with TS, and showed that the mean age of TS diagnosis was 6.6 years [8], which was a significant improvement compared to the results that Massa found over 10 years prior [8, 9]. This study was also based in Belgium with a sample size of 100 patients and data was collected from 1972 to 1988 [9]. Ten years later, we have noted only marginal improvement in the age at diagnosis of TS (5.89 years).

Gravholt et al. noted that, in Denmark, there is an increase in TS diagnosis during the antenatal period [5]. There is no association between TS and increase in maternal age, but it is thought that this may be the reason for antenatal screening [5]. Our results showed that at least 29% of pre-natal diagnoses were secondary to maternal age. This figure could be higher but unfortunately we did not have all the reasons for screening in this group.

Following on from antenatal diagnosis, the next opportunity for diagnosis is the neonatal period. By using the ‘guidelines for screening for TS’ [7], three of the major features (webbed neck, peripheral lymphoedema and coarctation of the aorta) and several minor features can be potentially identified during the routine newborn examination. Education in this area could lead to appropriate screening soon after birth and optimal intervention can be given to those found to have the diagnosis of TS.

Interestingly, developments in TS showed that nearly 10% of patients, who were diagnosed during infancy and childhood, were found to have cardiovascular defects [6]. These defects include coarctation of the aorta and left heart hypoplasia [6]. Wong et al. completed a cohort study with a sample of 132 patients with known coarctation of the aorta [10]. It was concluded that 5.3% of females with coarctation of the aorta were diagnosed with TS following routine karyotype analysis. Their recommendation was that all female patients with coarctation of the aorta should have TS screening immediately after diagnosis [10]. Of those with cardiovascular disease, many present with aortic dissection or aneurysm, which can be fatal [6]. Therefore identifying cardiovascular defects as early as possible is important so that the patients can be managed and monitored appropriately. Also, it would allow early education and advice to the girl and her family regarding the disease and symptoms to be aware of [6].

The Dutch height screening guidelines showed an increase in referrals with short stature especially with inaccuracy of length measurements in the first 3 years of life [11]. In this study, the authors felt that the then proposed UK consensus approach could lead to less referral but was not sensitive enough to detect TS promptly. The authors concluded that a scheme is required which has high sensitivity and low false positive results [11]. Another paper reviewed multiple studies worldwide and showed that a number of pathological conditions (including TS) were diagnosed where short stature was the only clinical finding. Therefore, it has highlighted that without height screening programmes in these countries many patients would have had a delay in their diagnosis [12]. Early detection means affected children can have optimal management and improve their quality of life [12]. There is no community-screening program dedicated to identify short stature at the moment in UK. Some auxology measurements are undertaken as part of the National Child Measurement Program with a focus on identifying obesity. However, there is no dedicated pathway to identify short stature from these [13].

Our study showed that the majority (54%) of the patients were diagnosed during the childhood period. 53% of this group was screened secondary to short stature. The guidelines recommend screening if a girl has an unexplained short stature (height < 5th percentile) [7]. The potential delay in diagnosis for these children is difficult to assess in the UK because there is no community height-screening programme. Prompt investigation of children with abnormal growth has been shown to give the best chance for effective treatment and thus good clinical outcomes [14]. It allows recombinant human growth hormone (GH) to be introduced in a timely fashion to improve the height prior to oestrogen therapy [4, 8]. If GH is started early the mean adult height is around 150 cm compared to 140 cm without GH [2]. If diagnosis is made after the age of twelve years, the time gap to initiate GH therapy is missed and this results in a lower adult height [8].

We found that 20% of patients were diagnosed between the ages of 12 and 18 years. In TS, 80% of the girls have delayed puberty [1], and in this study 15% of those presenting during adolescence were worried about delayed puberty. Guidelines state that all females who have not reached Tanner Stage 2 of breast development by age 13 or primary amenorrhoea by 15 years should be investigated. Unfortunately, often this problem is not acted on immediately and diagnosis can be delayed [1]. Puberty is induced by using oestrogen therapy and the timing and dosage is extremely important for each individual girl with TS. Early diagnosis of TS is essential so that GH can be given for the appropriate duration prior to initiating oestrogen therapy [4]. GH and oestrogen at the appropriate age has shown to give girls a near normal adult height, improved bone mass and sexual function [1].

Another issue that needs to be addressed for TS patients is initiating psychological and educational support to optimise social interaction and educational achievements. All of these factors can certainly improve the quality of life of those affected.

## Conclusion

We have shown in this retrospective study, that 48% of the girls with TS are still diagnosed only after the age of 5 years. Short stature triggered evaluation for most patients diagnosed in childhood and adolescence. Lack of community height-screening programme to identify children with short stature and lack of awareness could have led to potential delays in diagnosing TS. New strategies for earlier detection of TS and national guidelines are needed.

## Abbreviations

GH: Growth Hormone
TS: Turner Syndrome
